# Promotion of data sharing needs more than an emergency: An analysis of trends across clinical trials registered on the International Clinical Trials Registry Platform

**DOI:** 10.12688/wellcomeopenres.17700.1

**Published:** 2022-03-21

**Authors:** Laura Merson, Duduzile Ndwandwe, Thobile Malinga, Giuseppe Paparella, Kwame Oneil, Ghassan Karam, Robert F. Terry

**Affiliations:** 1 Infectious Diseases Data Observatory, University of Oxford, Oxford, OX3 7FZ, UK; 2 Cochrane South Africa, South African Medical Research Council, Cape Town, 7505, South Africa; 3 University of Exeter, Exeter, EX4 4PY, UK; 4 Sierra Leone Ministry of Health and Sanitation, Freetown, Sierra Leone; 5 World Health Organisation, Geneva, Switzerland; 6 Special Programme for Research and Training in Tropical Diseases (TDR), Geneva, Switzerland

**Keywords:** Data sharing; clinical trials, clinical trial registration; ICTRP, individual patient data, health research, COVID-19, SARS-CoV-2, public health emergencies

## Abstract

BACKGROUND: A growing body of evidence shows that sharing health research data with other researchers for secondary analyses can contribute to better health. This is especially important in the context of a public health emergency when stopping a pandemic depends on accelerating science.

METHODS: We analysed the information on data sharing collected by the 18 clinical trial registries included in the WHO International Clinical Trials Registry Platform (ICTRP) to understand the reporting of data sharing plans and which studies were and were not planning to share data. Data on sponsor and funder organisations, country of recruitment, registry, and condition of study were standardised to compare the sharing of information and data across these facets. This represents the first ever comprehensive study of the complete data set contained in ICTRP.

RESULTS: Across 132,545 studies registered between January 2019 and December 2020, 11.2% of studies stated that individual patient data (IPD) would be shared. Plans to share IPD varied across the 18 contributing registries– information on data sharing was missing in >95% of study records across 7/18 registries. In the 26,851 (20.3%) studies that were funded or sponsored by a commercial entity, intention to share IPD was similar to those that were not (11.5% vs 11.2%). Intention to share IPD was most common in studies recruiting across both high-income and low- or middle-income countries (21.4%) and in those recruiting in Sub-Saharan Africa (50.3%). Studies of COVID-19 had similar levels of data sharing to studies of other non-pandemic diseases in 2020 (13.7% vs 11.7%).

CONCLUSIONS: Rates of planned IPD sharing vary between clinical trial registries and economic regions, and are similar whether commercial or non-commercial agencies are involved. Despite many calls to action, plans to share IPD have not increased significantly and remain below 14% for diseases causing public health emergencies.

## Introduction

It is broadly recognised that scientific discovery is accelerated when health research data are made available to those outside of the original research team to combine with other data sets and/or use for secondary analyses. A growing body of research provides evidence that secondary analyses can contribute to better health ^1– 5^. When the research community has access to individual patient data (IPD) that underlie research results, new analyses can be done by other researchers with different ideas and expertise and data can be pooled for meta-analysis to increase statistical power. Enabling access to IPD makes the research results more transparent and trusted. The value of sharing data is in answering new research questions that could not be addressed by individual datasets or by the primary researchers alone. In recognition of this value, health institutions, research funders, publishers and the scientific community are increasingly requiring individual patient research data to be made accessible to other researchers for new analyses. This practice has been well established in the field of genomic research, where the depositing of genomic data into a public repository is a condition of most research funders. With a view to realising this value from the clinical data generated by research, new statements and updated policies promoting access to clinical data are regularly released. This is especially the case in the context of recent public health emergencies when expediting evidence on effective treatments and infection prevention can translate quickly into life-saving policy decisions ^6– 9^.

Advocacy for sharing of data during public health emergencies was catalysed following the collective failures in outbreak recognition, reporting and response during the 2013-2016 Ebola virus disease epidemic in West Africa ^10– 13^. The World Health Organization (WHO) held a consultation in 2015 on Developing Global Norms for Sharing Data and Results during Public Health Emergencies that resulted in a policy statement urging all international stakeholders “that timely and transparent pre-publication sharing of data and results during public health emergencies must become the global norm” ^14^. The same year, WHO launched the R&D Blueprint strategy for preparedness and activation of research during pandemics, specifically naming the diseases of focus in a list of priorities for research and development in emergency contexts ^15^. Subsequent emergencies triggered new statements including a 2017 joint statement on public disclosure of results from clinical trials that voices support for sharing research data where appropriate ^16^. Later, renewed encouragement for “all researchers to share their data as quickly and widely as possible” was issued following the 2020 declaration of a public health emergency of international concern (PHEIC) in response to the emergence of SARS-CoV-2 ^8^. These iterative instructions and prioritised diseases provide an unambiguous directive that must be upheld and monitored. However, measuring data sharing practices and trends across the research landscape to know whether progress is being made, is challenging.

One source of information on data sharing plans and mechanisms is the WHO International Clinical Trials Registry Platform (ICTRP). Established in 2006, ICTRP was launched as a response to the World Health Assembly mandate to “establish a voluntary platform to link clinical trials registers in order to ensure a single point of access and the unambiguous identification of trials ^17^." The platform consolidates 24 data fields from each of the clinical studies registered on 18 international registries that make up the ICTRP Registry Network ^18, 19^. These data fields, collectively called the WHO Trial Registration Data Set, include the information identified as most critical to make available to the global research community to increase transparency in clinical research ^18^. Each registry collects the 24 data fields required by ICTRP as well as other fields required by the registry according to their unique policies and guidance on registration ^20^.

The 24 ^th^ data field collects information on access to the individual patient data (IPD) from the research. In 2017, it was added to the WHO Trial Registration Dataset as an optional variable; recognising that access to IPD is important to maximise potential health improvements from the research and therefore that information about how to access the data would further increase transparency and science ^21^. The 24 ^th^ data field includes two sections: (1) a “statement regarding the intended sharing of de-identified individual clinical trial participant-level data (IPD)”, captured as a YES, NO or UNDECIDED response to the question: “Plan to share IPD?” and (2) a free-text field to address “what IPD will be shared, when, by what mechanism, with whom and for what types of analyses”, captured under the header: “Plan description.” UNDECIDED was later removed from the options, leaving only YES or NO as the current response options. In 2019, completion of the 24 ^th^ data field became a mandatory part of the WHO Registry criteria, meaning that all primary registries in the WHO Registry Network were required to submit this information on ICTRP for newly registered trials. This promotion to a mandatory data element aligned with the policy of the International Committee of Medical Journal Editors (ICMJE) that required publications reporting trials recruiting participants from 1 January 2019 onwards to include a data sharing statement in the trial registration ^21^.

As the ICTRP captures information from 18 international registries it provides a representative sample of the availability of IPD across the global clinical trials landscape and is a suitable resource to evaluate IPD sharing practices. Previous studies have examined sub-sets of data from ICTRP to evaluate data on certain diseases ^22^, individual registries ^23, 24^, specific geographic regions ^25^, medications ^26^, networks ^24^, and pre-2019 time periods ^23, 27, 28^. However, no previous study has undertaken an examination across the entire ICTRP dataset to show how this 24 ^th^ data field is being used, and the information it contains. At this early point in the development of data sharing practice and the capture of information on the topic, it is important to have a baseline understanding of how ICTRP can be used to monitor and measure progress. An understanding of how to improve the utility of ICTRP to capture and track this information accurately will inform the development of good practice.

Therefore, in order to build this evidence base we analysed the contents of the IPD sharing fields of the ICTRP database and how they differ across time, economic and geographic region, diseases relevant to public health emergencies, type of funder or sponsor, and registry.

## Methods

Following a request to WHO, we obtained the complete ICTRP dataset as of 15 December 2020 ^29^. The dataset included all 24 data fields submitted by 17 ICTRP primary registries plus the clinicaltrials.gov registry (see Table 1 for registries). Duplicate registrations were identified by bridging variables within the ICTRP dataset; these are assigned based on secondary identifiers in the registrations of trials registered more than once within a registry or on more than one registry ^30^. “Parent” registrations, as defined by those responsible for the trial, were considered the master record and retained; “child” registrations were removed to deduplicate the analysis dataset.

**Table 1.** Registries with data included on ICTRP.

WHO Primary Registries ● Australian New Zealand Clinical Trials Registry (ANZCTR) ● Brazilian Clinical Trials Registry (ReBec) ● Chinese Clinical Trial Registry (ChiCTR) ● Clinical Research Information Service (CRiS), Republic of Korea ● Clinical Trials Registry, India (CTRI) ● Cuban Public Registry of Clinical Trials (RPCEC) ● EU Clinical Trials Register (EU-CTR) ● German Clinical Trials Register (DRKS) ● ISRCTN ● Iranian Registry of Clinical Trials (IRCT) ● Japan Primary Registries Network (JPRN) ● Lebanese Clinical Trials Registry (LBCTR) ● Pan African Clinical Trial Registry (PACTR) ● Peruvian Clinical Trial Registry (REPEC) ● Sri Lanka Clinical Trials Registry (SLCTR) ● Thai Clinical Trials Registry (TCTR) ● The Netherlands National Trial Register (NTR) ICTRP Data Providers ● Clinicaltrials.gov (CT)

WHO Primary Registries and Data Providers with data included in ICTRP and in this analysis

All data coding was performed by two independent researchers based on predetermined definitions described in Table 2. The results of the duplicate coding were compared and discrepancies between the assigned codes were identified. Discrepancies were resolved by one of the researchers based on review of the source data, coding definitions, and a search for additional information (e.g. the company website) to determine which definition outlined in Table 2 was correct. Where additional evidence could not resolve a conflict, the final coding was agreed between two researchers based on closest fit with the definitions.

**Table 2.** Database coding definitions.

ECONOMIC DATA	
High-income countries (HIC)	Coded based on the World Bank Lending Groups data (June 2020) according to the World Bank Atlas Methodology ^31, 32^. Includes those classified as High income only. For a full list of countries, see Extended Data 1 ^29^.
Low- and middle-income countries (LMIC)	Coded based on the World Bank Lending Groups data (June 2020) according to the World Bank Atlas Methodology ^31, 32^. Includes those classified as Low income, Lower middle income, and Upper middle income. For a full list of countries, see Extended Data 1 ^29^.
**GEOGRAPHIC DATA**	
East Asia & Pacific	Coded based on the World Bank Country Groups (June 2020) according to the World Bank Atlas Methodology ^31, 32^. For a full list of countries in each category, see Extended Data 1 ^29^.
Europe & Central Asia	
Latin America & Caribbean	
Middle East & North Africa	
North America	
South Asia	
Sub-Saharan Africa	
**CONDITION / DISEASE DATA** Priority diseases are defined by WHO as those which pose the greatest public health risk due to their epidemic potential and/or those for which there is no or insufficient countermeasures. These are listed on the WHO list of priority diseases for research and development in emergency contexts ^33^. Priority diseases which have triggered declaration of a PHEIC were coded as individual diseases. Priority diseases which have not triggered a PHEIC were grouped.	
COVID-19	Condition data field contains any terms indicative of COVID-19
Zika	Condition data field contains any terms indicative of Zika virus
Ebola	Condition data field contains any terms indicative of Ebola virus disease
Other WHO priority pathogens	Condition data field contains any terms indicative of Crimean-Congo haemorrhagic fever, Marburg virus disease, Lassa fever, Middle East respiratory syndrome coronavirus, Severe Acute Respiratory Syndrome, Rift Valley fever, Nipah or henipaviral diseases.
Other condition	Condition fields that did not contain any terms related to the diseases above.
**FUNDER AND SPONSOR DATA** Each sponsor and funder were individually searched on the internet to determine the status, registration, type, mission, structure, remit and/or links of the organisation/institution. Study registrations are coded overall as ‘Commercial’ if any of the sponsors or funders are classified as ‘Commercial’. Those with no details of any sponsor or funder were excluded from analysis.	
Commercial	For organisations where evidence of profit-driven corporate mission or company structure was identified.
Non-commercial	For organisations where evidence of non-profit status was identified, including governments, foundations, academic and research institutions, health care provision facilities, and public health agencies.

Definitions used for coding ICTRP economic, geographic, condition and funder/sponsor data for the purpose of this analysis.

A descriptive analysis of the data across time, country income group, region, sponsor and funder type, priority disease status and IPD availability plan was prepared for all studies registered in 2019 or 2020 using Microsoft Excel.

A fixed set of details of the registries which provide data to ICTRP were collected by searching the individual registry websites, ICTRP website, academic literature and grey literature. A questionnaire was sent to the administrators of each registry to confirm and supplement the information identified, responses were received from 12 of the 18 registries (Australian New Zealand Clinical Trials Registry; Brazilian Clinical Trials Registry; Clinical Research Information Service, Republic of Korea; Clinicaltrials.gov; Cuban Public Registry of Clinical Trials; German Clinical Trials Register; ISRCTN; Iranian Registry of Clinical Trials; Pan African Clinical Trial Registry; Peruvian Clinical Trial Registry; Sri Lanka Clinical Trials Registry; Thai Clinical Trials Registry). Details of the information collected is available in Underlying Data – Registry Information ^29^.

## Results

The ICTRP dataset included 643,414 clinical study registrations as of 15 December 2020. Following the removal of duplicate records, 593,595 study registrations were included in the final analysis. 132,545 (22.3%) of these registrations were made in 2019 or 2020 when completion of information about IPD availability was mandatory.

### Increasing availability of information on IPD sharing plans

**Figure 1.**
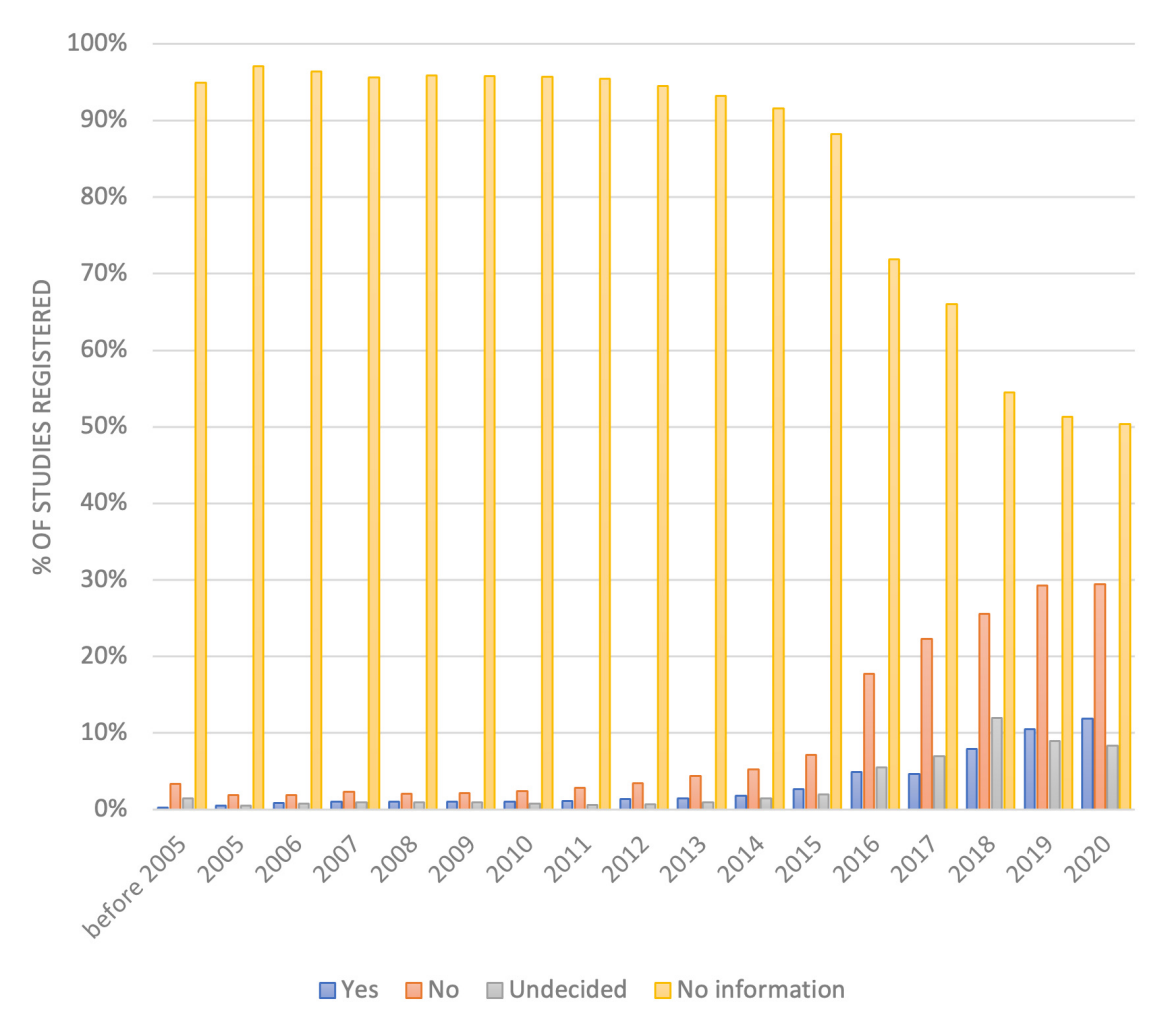
IPD sharing plans of all studies included on ICTRP. Plans to share IPD from each registered study are listed as Yes, No or Undecided. Availability of information increases from 2017 onwards.

Across all records in ICTRP, 143,282/593,595 (24.1%) study registrations have completed the 24 ^th^ data variable, providing information on IPD sharing plans. 28,684/143,282 (20.0%) of these studies planned to share IPD and 86,188/143,282 (60.1%) planned to not share IPD. In the studies registered after the 1 January 2019 mandate for completion of the 24 ^th^ data field, 65,188/132,545 (49.2%) have information on IPD sharing plans, of which 14,854/65,188 (22.8%) plan to share IPD and 38,892/65,188 (59.7%) plan to not share IPD ( Figure 1). Results across ICTRP show increasing completion of the data field from its introduction in 2017 and low levels of retrospective completion in studies registered before 2017.

### Wide distribution of study records and IPD sharing plans across registries

**Figure 2.**
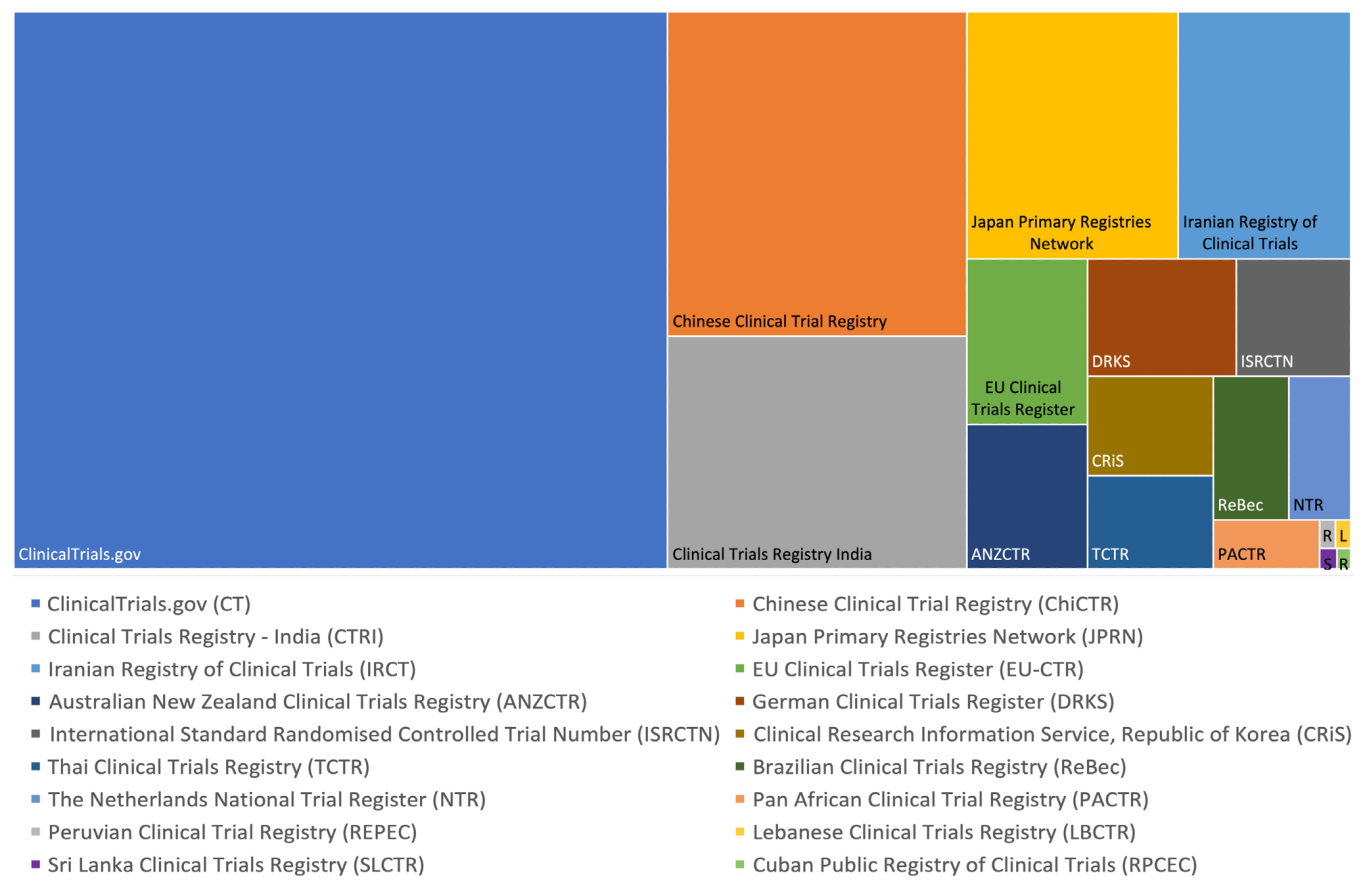
Distribution of study registrations across the ICTRP Registry Network 2019-2020. The number of studies registered in 2019 or 2020 for each registry contributing to ICTRP is shown as a volume, relative to other registries and the total number.

Study registrations made since January 1 ^st^ 2019, are unevenly distributed across 18 source registries with 48.9% (64,809/132,545) originating from clinicaltrials.gov. The next largest volumes of trials are registered in the Chinese Clinical Trial Registry (13.0%), Clinical Trials Registry India (9.4%), Japan Primary Registries Network (7.0%) and the Iranian Registry of Clinical Trials (5.7%). Each of the 13 other registries hold less than 3% of all studies on ICTRP (range 0.0% - 2.7%) (51-3558/132,545) ( Figure 2 and Extended Data 2 ^29^).

Availability of information on IPD sharing ranges from 0% to 100% across the 18 registries (IQR 0.6-99.6%). Up until December 15 ^th^, 2020, many registries had provided little or no information on IPD availability to ICTRP. This information was available in 0% of 2019 and 2020 study registrations submitted by Clinical Trials Registry - India, EU Clinical Trials Register, and The Netherlands National Trial Register, and in less than 5% of registrations made on Brazilian Clinical Trials Registry, Chinese Clinical Trial Registry, Japan Primary Registries Network and Thai Clinical Trials Registry. Across the 11 other registries, the proportion of studies reporting information on IPD sharing ranged from 65.0% to 100% with (IQR 90-100%).

**Figure 3.**
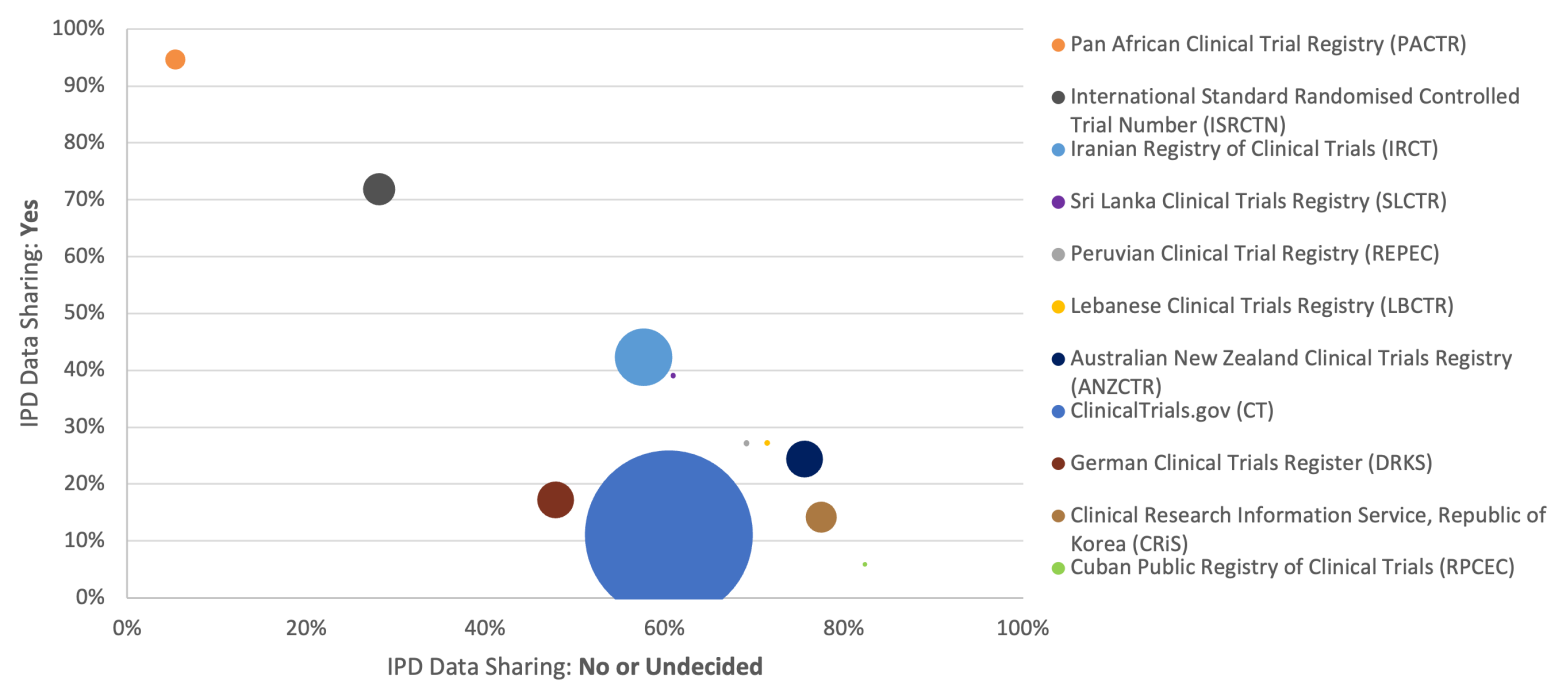
Proportion of studies with intention to share IPD varies across registries 2019-2020. The proportion of studies in each registry planning to share IPD is plotted against those not planning or undecided on IPD sharing (x and y axes respectively). The size of the circle indicates the relative volume of studies on each registry.

The proportion of studies registered as planning to share IPD ranged from 5.9% on Cuban Public Registry of Clinical Trials with 3/51 studies to 94.6% on Pan African Clinical Trial Registry with 882/932 studies (IQR 15.7-40.7%) ( Figure 3 and Extended Data 3 and 4 ^29^).

### Variation in plans to share IPD across geographic and economic regions

The number of studies recruiting participants per geographic region varies greatly, as do the plans for IPD data sharing in each region. Intention to share IPD was reported in 9.2% (7,890/85,745) of studies recruiting participants only in high-income countries (HICs), 16.2% (5,464/33,770) of studies recruiting only in low- and middle-income countries (LMIC) and 21.4% (518/2,419) of studies recruiting in both HICs and LMICs. When examined by geographic region, the lowest levels of IPD sharing were planned for studies recruiting in South Asia (2.7%, 427/16,098) and Latin America & Caribbean (2.9%, 622/4,830). Those recruiting in the Middle East & North Africa (33.7%, 4,252/12,624) and Sub-Saharan Africa (50.3%, 869/1,726) reported the highest levels of IPD sharing planned. Table 3 shows the number of studies recruiting participants and their IPD sharing plans per economic and geographic grouping.

**Table 3.** IPD sharing plans per economic and geographic region.

	IPD sharing plans stated	IPD sharing: YES	IPD sharing: NO	IPD sharing: UNDECIDED
All studies	65,188 / 132,545 (49.2%)	14,854 (11.2%)	38,892 (29.3%)	11,442 (8.6%)
Studies recruiting in HICs * only	41,818 / 85,745 (48.8%)	7,890 (9.2%)	28,090 (32.8%)	5,838 (6.8%)
Studies recruiting in LMICs ** only	15,560 / 33,770 (46.1%)	5,464 (16.2%)	5,774 (17.1%)	4,322 (12.8%)
Studies recruiting in both HICs & LMICs	976 / 2,419 (40.3%)	518 (21.4%)	350 (14.5%)	108 (4.5%)
South Asia	1,435 / 16,098 (9.0%)	427 (2.7%)	781 (4.9%)	227 (1.4%)
Latin America & Caribbean	1,886 / 4,830 (29.1%)	622 (2.9%)	983 (20.4%)	281 (5.8%)
East Asia & Pacific	12,279 / 42,769 (28.7%)	2,489 (5.8%)	8,219 (19.2%)	1,571 (3.7%)
Europe & Central Asia	19,242 / 31,493 (61.1%)	4,129 (13.1%)	11,614 (36.9%)	3,499 (11.1%)
North America	15,457 / 21,887 (70.6%)	3,292 (15.0%)	10,772 (49.2%)	1,393 (6.4%)
Middle East & North Africa	10,760 / 12,624 (85.3%)	4,252 (33.7%)	2,935 (23.2%)	3,573 (28.3%)
Sub-Saharan Africa	1,295 / 1,726 (75.0%)	869 (50.3%)	333 (19.3%)	93 (5.4%)
Country / region unknown	6,834 / 10,611 (64.4%)	982 (9.2%)	4,678 (44.1%)	1,174 (11.1%)

Number of studies in each economic and geographic region that state IPD sharing plans, and the contents of those plans (Yes, No, or Undecided) *HICs – high-income countries **LMICs – low- and middle-income countries HIC, LMIC, and HIC & LMIC categories are mutually exclusive. Geographic regions are not exclusive as many studies are run in more than one region.

### Similar levels of IPD sharing between commercial and non-commercial sponsors and funders

**Figure 4.**
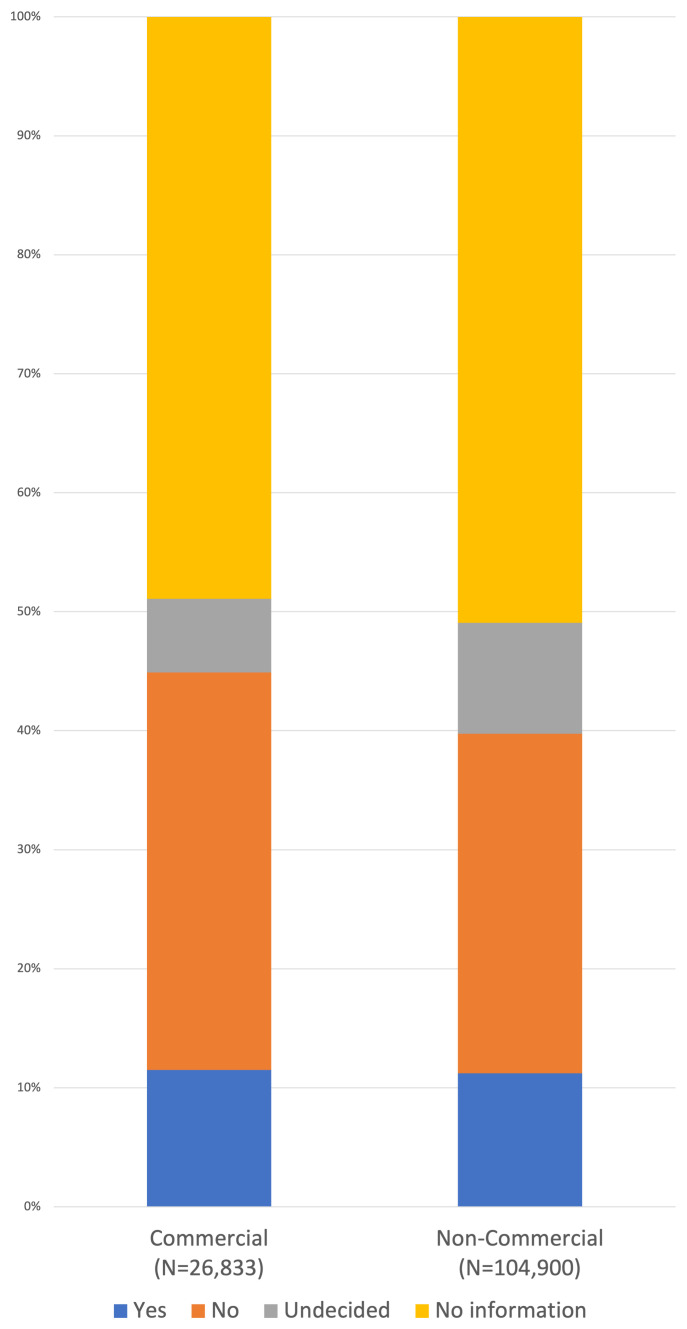
IPD sharing plans are similar between commercial and non-commercial sponsors and funders. Plans to share IPD are compared between studies sponsored or funded by a commercial entity, and studies that are not sponsored or funded by a commercial entity. Plans are recorded as Yes, No or Undecided where information is available.

A fifth (26,833/132,585; 20.2%) of all studies registered after the start of 2019 declare the involvement of an entity with commercial interests as the sponsor and/or funder. 13,706/26,833 (51.1%) of these registrations had information on plans to share IPD available, 3,082 (11.5%) state ‘Yes’, IPD will be shared and 8,968 (33.4%) said IPD would not be shared. Of the 104,900/132,585 (79.1%) studies that do not list a commercial entity among the funders or sponsors, 51,465/104,900 (49.0%) have information on IPD sharing, or which 11,765 (11.2%) said that IPD will be shared and 29,918 (28.5%) said that IPD would not be shared ( Figure 4). 812 studies listed no funders or sponsors.

### Small difference in IPD sharing on pathogens causing public health emergencies

**Figure 5.**
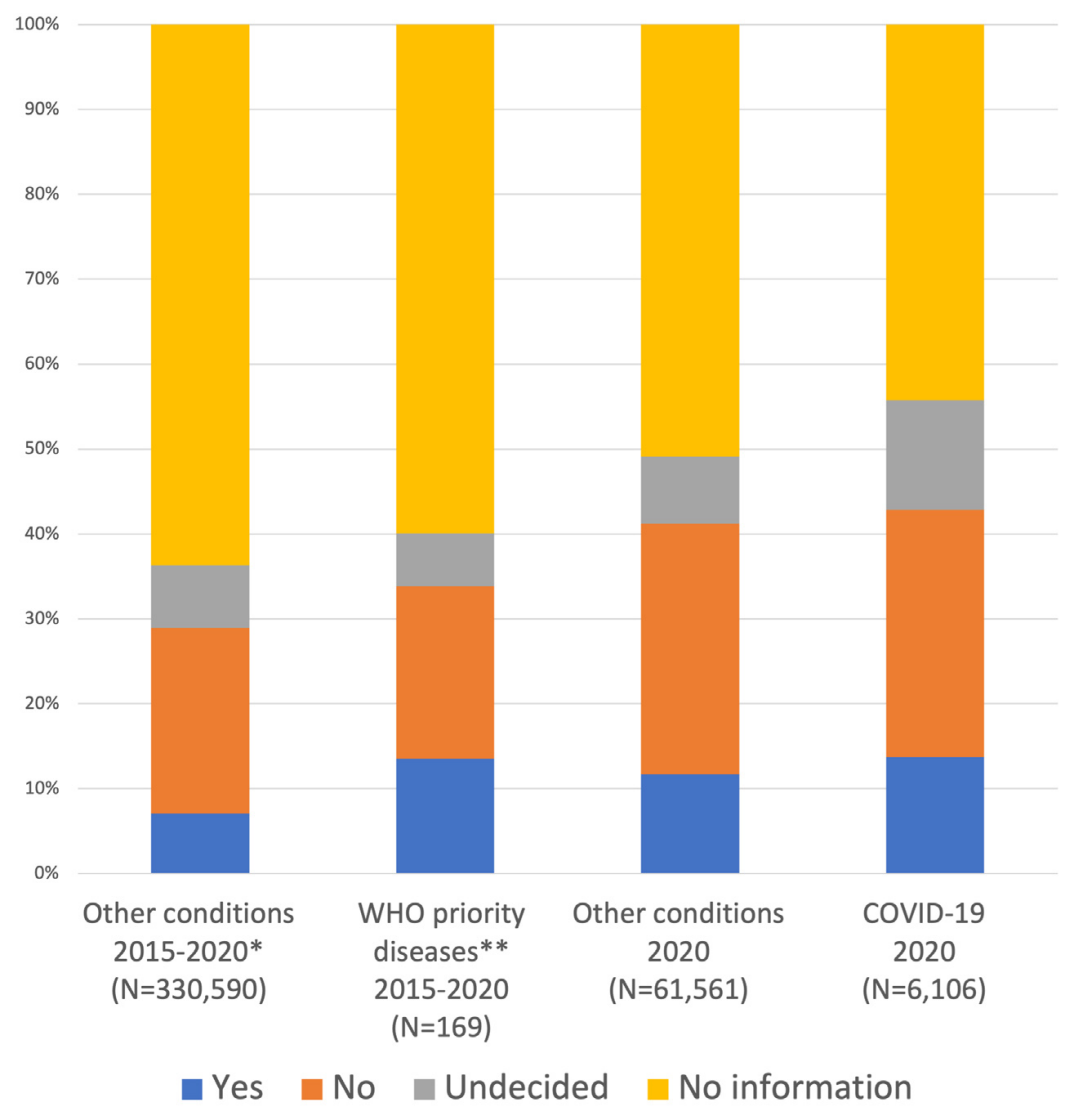
Little difference in plans to share IPD from studies of WHO priority diseases compared to other diseases of the same period. Plans are recorded as Yes, No or Undecided where information is available. Two left bars - Plans to share IPD are compared between studies of diseases prioritised by WHO for research and development in emergency contexts 2015-2020, and studies of other conditions in the same period. *Annual distribution adjusted to match that of WHO priority diseases. ** Excluding COVID-19. Two right bars - Plans to share IPD are compared between studies of COVID-19 in 2020, and studies of other conditions in 2020.

A small fraction of studies focused on diseases prioritised by WHO for research and development in emergency contexts, including: COVID-19, Crimean-Congo haemorrhagic fever, Marburg virus disease, Lassa fever, Middle East respiratory syndrome coronavirus (MERS-CoV), Severe Acute Respiratory Syndrome (SARS), Nipah and henipaviral diseases, and Rift Valley fever ^33^. Of these diseases, COVID-19 had the highest number of studies registered (6,108/593,595) representing 1.0% of the entire database and 9.0% of all studies registered in 2020. Just over half of these studies (3,401/6,108; 55.7%) provided information on IPD sharing. 13.7% of studies reported that IPD would be shared, while 29.1% reported that IPD would not be made available and 12.9% were undecided about IPD sharing. These results are similar to those of non-priority diseases registered in 2020 where information was available in 49.1% (30,221/61,561), 11.7% reported that IPD would be shared, 29.5% reported that IPD would not be shared and 7.9% were undecided ( Figure 5).

In addition to COVID-19 research, 169 studies on other priority diseases were registered within the WHO Registry Network since 2015 when the WHO Blueprint R&D was launched. This is 0.05% of all studies registered in this period. Information on IPD sharing was available in 73/169 (43.2%) of studies on priority diseases. Plans to share IPD were confirmed in 13.6% of studies, plans to not share IPD were reported in 22.5% of studies and 7.1% were undecided. In the non-priority diseases registered from 2015-2020, there was slightly less availability of information on IPD sharing (126,240/330,590; 38%), with 7.1% of studies planning to share IPD, 21.9% planning to not share IPD and 7.4% undecided ( Figure 5).

### Limitations

Accuracy of the data on the ICTRP registry is reliant on the completion and accuracy of information by those registering the trial, any quality assurance processes applied by the primary registry, and the completeness of data transfer from the primary registry to ICTRP. Individual registries each implement their own approach to standardising the data they host. Controlled vocabularies (i.e., fixed terms to standardise how the same thing is recorded across the registry) and predefined categories to capture information such as conditions of study, funder and sponsor organisations, or type of study, vary across the registries. To aggregate information across these varied sources, ICTRP has implemented a flexible data model to accommodate the variety of content. This flexibility limits the feasibility of ICTRP to apply quality assurance measures to the volume of data it receives. The result is an aggregated dataset with fields that include a variety of terms to mean the same thing. For example, among the 6,106 studies of SARS-CoV-2 infection included in this dataset, we identified 2,492 unique entries in the variable for condition of study. Such variety makes robust analysis challenging as terms of interest are difficult to identify.

Though the WHO Trial Registration Data Set’s 24 ^th^ data field on plans to share IPD was introduced in 2017 and made mandatory in 2019, completion rates remain low for many of the registries providing data. There are several reasons for the missing data, including differences in the study registration policies and data transfer procedures across the registries. While most registries require information about IPD data sharing to be entered at the time of registration, the Lebanese Clinical Trials Registry requires this information before participant recruitment ^34^, ClinicalTrials.gov and the Brazilian Clinical Trials Registry require this information to be submitted on study completion ^35, 36^, and The Peruvian Clinical Trials Registry does not currently have a policy that requires this information ^37^. These differences introduce the possibility of a lag between trial registration and the availability of the information. Furthermore, the process of updating the 24 ^th^ data field is not consistent across all registries. The frequency of data update varies between registries, and some registries do not update data on the 24 ^th^ variable. This practice means that trials with no data entered in the 24 ^th^ field at initial registration, may not be updated with details added later in the life of the trial. To ensure the accuracy and recency of information, data should be extracted directly from each registry, though this is operationally challenging as few of the registries offer data download or API access.

Identifying duplicate registrations for the same clinical trial in different registries is an additional challenge in the ICTRP dataset. Many researchers register their studies in more than one registry to meet the requirements of international funders, sponsors and sites. A bridging variable exists in the ICTRP data model to identify and link studies registered across multiple registries. However, previous studies have demonstrated the incompleteness of this identification and linking processes, and the presence of unidentified duplicates within the ICTRP dataset ^38^. Only duplicates identifiable by these bridging variables were removed from the data in this analysis.

## Discussion

While there is broad recognition of the value of sharing IPD from research and development, intention to make IPD available continues to be low across the global community of investigators registering clinical studies. Rates of planned IPD sharing vary between clinical trial registries and economic regions and are similar whether commercial or non-commercial agencies are involved. Plans to share IPD have not been significantly impacted by declarations of public health emergencies. Despite many calls to action, plans to share IPD have not increased significantly for diseases causing public health emergencies and remain below 14% of registered trials. Understanding the factors that impact investigator ability and willingness to share IPD can support planning and policy to encourage the availability of IPD where the biggest gaps occur.

### Registries have a key role in access to information

Several stakeholders have a role in promoting access to information about data sharing. Several research funders have developed policies that require data management plans to detail how data will be shared in advance of patient recruitment to the research they fund, including some of the world’s largest such as the US National Institutes of Health ^39, 40^, the European Commission ^41^, Wellcome ^42^, and the Bill & Melinda Gates Foundation ^43^. Many journals have policies that mimic the ICMJE policies requiring registration of clinical trials they publish, and a data sharing statement to be included in the trial registration ^21^. Regardless of who requires the availability of this information, it is the clinical trial registries that accept, structure, and host the information. Each WHO Primary Registry includes registration variables that comply with the WHO Trial Registration Data Set. However, the policies on completion of this information and procedure for transfer of the information to ICTRP vary. 6/7 registries with >95% completion of IPD information had a policy for mandatory completion of these variables from 2019 or prior. 1/7 registries that had <5% completion of IPD information mandated completion of these variables, 3/7 had optional completion and 3/7 had no guidance on any requirement. None of these had evidence of a policy in place by 2019. Additionally, three of these registries had not transferred any data on these variables to ICTRP as of December 2020. (See Underlying Data - Registry Information for details of individual registry policies and procedures ^29^).

It is difficult to assess the impact of registry policies on the likelihood of researchers being willing to share IPD. The highest rates of willingness to share IPD (24.4-94.6% yes for IPD sharing) occur in the seven registries with reporting rates >95%. Lower rates of willingness to share IPD in other registries are difficult to interpret due to large amounts of missing information.

### Economic and geographic differences may have many drivers

There are differences in data sharing plans across regions grouped by geography or income levels. The high levels of data sharing reported in studies recruiting in Sub-Saharan Africa align with the high levels reported in the Pan-African Clinical Trial Registry. These may be attributable to the policies of the funders supporting the research, national legislation, the policies of PACTR, research culture, or other influences.

Similarly, further research is needed to understand why the highest rates of data sharing intentions are seen in studies recruiting in both HICs and LMICs, and why studies recruiting in LMICs plan to share data more often than studies recruiting in HICs.

### Sponsor and funder policies

Research funders and sponsors, and the policies they apply to the research they support, have a key role in promoting data sharing. However, reports on the implementation and impact of these policies show that translation of these policies into data access can be challenging across both industry and academia ^44– 48^. Willingness to share IPD was found to be similar between studies that did or did not have a commercial funder or sponsor involved (11.5% vs 11.2%). Though funder policies have been found to be key determinants of data sharing practice, many other drivers and barriers contribute to the final decision on whether data are shared ^49, 50^.

### The call to arms for data sharing in emergencies is not being heard

Perceived failures in the global response to public health emergencies since the 2013-2016 Ebola virus disease outbreak have rallied the health community to call for rapid sharing of research data to combat public health emergencies ^6, 8, 10, 51^. Statements and funding policies written in response to Ebola, Zika virus and, more recently, the COVID-19 pandemic have all emphasised the imperative to share data ^7, 9^. The 32 major funders that form the membership of the Global Research Collaboration for Infectious Disease Preparedness (GloPID-R) ^52^ have issued policies and resources targeting rapid access to data in health emergencies ^53^.

Despite these efforts and investments, the ICTRP data show no significant change in plans to share IPD for outbreak diseases versus other diseases were seen at the launch of the latest three PHEICs. Declaration of a public health emergency has been associated with rapid increases in academic output on the responsible disease ^54^. This surge in academic value of the data may decrease the likelihood of sharing as the perceived potential loss is greater for the data holder ^55^. As the WHO develops the new Pandemic Preparedness Framework and moves toward a paradigm of data as a global public good ^56^, incentives, protections and mandates that defeat the barriers to sharing must be addressed.

### Room for improvement

Though data limitations exist, the ICTRP is a valuable resource to monitor trends in clinical trial data sharing. The inclusion of information on IPD availability in the ICTRP registry enhances research transparency and helps to realise the scientific potential of access to IPD. This information would become more findable and usable if data could be standardised across registries. Encouraging alignment of controlled vocabularies such as SNOWMED ^57^ for conditions of study and Anatomical Therapeutic Chemical (ATC) ^58^ codes for medications would greatly improve the analysability of the dataset and open opportunity for understanding trends in the global clinical trials landscape. Implementing use of unique identifiers such as Crossref funder ID ^59^ and Research Organisation Registry (ROR) ID ^60^ would enable tracking of investment and compliance with data sharing policies by funders and institutions. The ICTRP has served as a convenor of standards across the primary registries and supported alignment of information and policies between them. This function should be further enabled to support quality standards and control so that this resource can be more readily mined to learn from the history and trends in clinical trials. Other studies have identified additional areas for improvement of the quality of data in ICTRP and its primary registries ^61^.

Registration of all clinical trials, including completion of the full WHO Trial Registration Data Set, is an ethical and regulatory requirement of conducting a clinical trial. Registries should support communication and compliance with this mandate by auditing the completion of all registration variables and sharing of full datasets with the ICTRP. Resources to explain the meaning of the IPD sharing variables are needed to support researchers to achieve the sharing plans they indicate in the registry record.

## Conclusions

ICTRP is an important resource for clinical trials transparency. Improvements to the quality, completion and standardisation in the registries that supply data to ICTRP are important to strengthening the integrity of this resource and the science that it supports. Auditing to ensure that trial registrations include all 24 essential elements of the WHO Trial Registration Data Set would be a valuable first step to quality improvement and should be the responsibility of the registries accepting the registrations.

Realising the health benefits of data sharing, particularly in the context of a public health emergency, can only occur when IPD from all clinical studies are made available to the research community. Mechanisms to protect the interests of researchers conducting studies must be established to reduce the barriers to data sharing and ensure fair distribution of the benefits. Funders, sponsors, journals, health agencies and researchers share the responsibility to maximise the impact of the research investment by promoting data sharing and increasing the availability of IPD. It is clear from this review of data sharing information and intentions, that there is room for improvement.

## List of abbreviations

ICMJE - International Committee of Medical Journal Editors

ICTRP – International Clinical Trial Registry Platform

IPD - individual patient data

PHEIC - Public Health Emergency of International Concern

WHO – World Health Organization

## Data availability

### Underlying data

Harvard Dataverse - Replication Data for: Promotion of data sharing needs more than an emergency: An analysis of trends across clinical trials registered on the International Clinical Trials Registry Platform. DOI: https://doi.org/10.7910/DVN/2ZNIFV ^29^

This project contains the following underlying data:

-    ICTRP Analysis Dataset (Curated analysis dataset to reproduce the manuscript results)

-    ICTRP download all registries but CT 15DEC20 (Raw data from ICTRP including data from all primary registries - not including data from clinicaltrials.gov)

-    ICTRP download CT registry 15DEC20 (Raw data from ICTRP including only clinicaltrials.gov)

-    Registry Information

-    International_Standards_for_Clinical_Trial_Registration_2018.pdf (Documentation for the ICTRP Dataset. The data dictionary for the raw ICTRP data is on page 23

### Extended data

This project contains the following extended data:

-    Extended Data 1-4 - Countries in each geographic region table, Number of studies registered table, Intention to share IPD table, Intention to share IPD graph

All data and materials are available under the terms of the Creative Commons Zero "No rights reserved" data waiver (CC0 1.0 Public domain dedication).
