# Supplementary material for: Promotion of data sharing needs more than an emergency: An analysis of trends across clinical trials registered on the International Clinical Trials Registry Platform
Source: Wellcome Open Res. 2022 Mar 21;7:101. [Version 1] doi: 10.12688/wellcomeopenres.17700.1 (PMC8980676; doi:10.12688/wellcomeopenres.17700.1)
Supplement: Peer review history

# Peer review: Reviewer response for version 1

**DOI:** 10.21956/wellcomeopenres.19585.r49342

**Published:** 2022-04-04

**Authors:** Oommen John

**Affiliations:** 1 George Institute for Global Health, University of New South Wales, New Delhi, Delhi, India

The authors highlight a significant global health challenge that persists despite well meaning efforts to improve compliance to clinical trial data sharing. The analysis highlights that a public health emergency of COVID-19 pandemic magnitude has not changed prevailing practices across commercial and academic clinical trials. 

 A key limitation of the study was the data on IPD sharing was not mandatory prior to 2019 and therefore only 24% of the study registrations had complete information on the same, whereas IPD sharing plans were available for 49.2% for the period after January 2019. Another limitation is the variability of the comprehensiveness of the data across different source registries and the non availability of IPD sharing details in many of these registries for the period till December 2020.

 Despite these limitations, the study offers valuable insights into key challenges that pertain to IPD sharing. Policy frameworks around public health emergencies does not seem to have any significant impact on IPD sharing and therefore forthcoming policy interventions such as the pandemic treaty negotiations need to consider the missed opportunities and economic impact of non availability of IPD that could influence clinical management guidelines and timely public health advisories.

Is the work clearly and accurately presented and does it cite the current literature?

Yes

If applicable, is the statistical analysis and its interpretation appropriate?

Yes

Are all the source data underlying the results available to ensure full reproducibility?

Yes

Is the study design appropriate and is the work technically sound?

Yes

Are the conclusions drawn adequately supported by the results?

Yes

Are sufficient details of methods and analysis provided to allow replication by others?

Yes

Reviewer Expertise:

Clinical trials, Public health research, data governance and digital health policy.

I confirm that I have read this submission and believe that I have an appropriate level of expertise to confirm that it is of an acceptable scientific standard.

# Peer review: Reviewer response for version 1

**DOI:** 10.21956/wellcomeopenres.19585.r49341

**Published:** 2022-03-23

**Authors:** Sharon Kaur Gurmukh Singh

**Affiliations:** 1 Faculty of Law, University of Malaya, Kuala Lumpur, Malaysia

This is an important study which sets out the current available information on data sharing plans as included in the ICTRP. Although many parties, including the WHO have consistently highlighted the importance of data sharing in the context of a public health emergency, there is little evidence of this in practice. This study provides a robust analysis of of data sharing practices across 18 clinical trial registers and highlights a number of steps that may be taken to improve both reporting of these practices as well as areas in which further research is urgently needed to understand and address this problem.

Is the work clearly and accurately presented and does it cite the current literature?

Yes

If applicable, is the statistical analysis and its interpretation appropriate?

Yes

Are all the source data underlying the results available to ensure full reproducibility?

Yes

Is the study design appropriate and is the work technically sound?

Yes

Are the conclusions drawn adequately supported by the results?

Yes

Are sufficient details of methods and analysis provided to allow replication by others?

Yes

Reviewer Expertise:

Research ethics

I confirm that I have read this submission and believe that I have an appropriate level of expertise to confirm that it is of an acceptable scientific standard.
